# DEMA: A Deep Learning-Enabled Model for Non-Invasive Human Vital Signs Monitoring Based on Optical Fiber Sensing

**DOI:** 10.3390/s24092672

**Published:** 2024-04-23

**Authors:** Qichang Zhang, Qing Wang, Weimin Lyu, Changyuan Yu

**Affiliations:** 1 Department of Electrical and Electronic Engineering, The Hong Kong Polytechnic University, Hong Kong; qichang.zhang@connect.polyu.hk (Q.Z.);; 2 Shenzhen Research Institute, The Hong Kong Polytechnic University, Shenzhen 518057, China

**Keywords:** vital signs monitoring, optical fiber sensor, DEMA, LSTM, MZI, EMD

## Abstract

Optical fiber sensors are extensively employed for their unique merits, such as small size, being lightweight, and having strong robustness to electronic interference. The above-mentioned sensors apply to more applications, especially the detection and monitoring of vital signs in medical or clinical. However, it is inconvenient for daily long-term human vital sign monitoring with conventional monitoring methods under the uncomfortable feelings generated since the skin and devices come into direct contact. This study introduces a non-invasive surveillance system that employs an optical fiber sensor and advanced deep-learning methodologies for precise vital sign readings. This system integrates a monitor based on the MZI (Mach–Zehnder interferometer) with LSTM networks, surpassing conventional approaches and providing potential uses in medical diagnostics. This could be potentially utilized in non-invasive health surveillance, evaluation, and intelligent health care.

## 1. Introduction

### 1.1. Background

It is universally acknowledged that human vital signs are of paramount importance in the realm of medical diagnosis. The ability to accurately and swiftly diagnose medical conditions not only significantly enhances the rate of successful treatment but also substantially reduces the financial burden associated with healthcare. However, a majority of healthcare institutions at present are heavily reliant on direct contact methods for testing, such as punctures and radiographs.

This traditional approach to testing, however, is fraught with two conspicuous drawbacks. Firstly, it is a resource-intensive and costly process. In most instances, patients can only be monitored within the confines of a hospital setting due to the need for specialized equipment and trained personnel. This not only limits the accessibility of such diagnostic methods but also places a significant strain on healthcare resources.

Additionally, the necessity for an uninterrupted link between the monitoring device and the individual being monitored may considerably limit their mobility and elevate their level of unease. This could conceivably result in diminished adherence from patients and adversely affect the precision of the diagnostic outcomes.

In recent years, a plethora of non-invasive monitoring techniques have emerged, many of which utilize laser sensing. While these methods have significantly improved patient comfort, they often compromise on accuracy, a critical factor in medical diagnosis. One of the primary objectives of our research is to optimize the accuracy of non-invasive monitoring techniques without sacrificing patient comfort.

Previously, a group headed by I. Smith assessed the repeatability of respiratory rate measurements utilizing the Bland–Altman technique [1]. Simultaneously, a team under R. Favilla introduced an innovative approach for tracking human heart rate [2]. These groundbreaking investigations have laid the foundation for the evolution of non-invasive physiological parameter monitoring, offering fresh perspectives and opportunities for subsequent studies.

Motivated by these pioneering research efforts, our group has developed a system that operates without direct contact, utilizing a micro-bend fiber optic sensor. This novel strategy aids in reducing the cost of the system and markedly improves the effectiveness of signal detection.

Compared to most conventional methods, our approach offers a substantial improvement in terms of production cost and process. By eliminating the need for direct contact, our system also significantly reduces patient discomfort and allows for greater mobility during testing.

In conclusion, our research aims to revolutionize the field of medical diagnosis by developing a cost-effective, efficient, and non-invasive monitoring system. By building on the work of previous researchers and incorporating innovative technologies, we hope to significantly improve the accuracy and comfort of medical testing, ultimately leading to better patient outcomes and a more sustainable healthcare system.

### 1.2. Progress of Research at the Current Stage

The integration of deep learning and fiber optic sensor technology to create a non-invasive vital sign signal monitoring model is an emerging field of research. This innovative approach aims to enhance the accuracy and efficiency of vital sign signal monitoring, thereby revolutionizing the healthcare industry.

Deep learning, a subset of artificial intelligence, is a technique that enables automatic recognition and understanding of patterns within large datasets [3]. It achieves this by learning from vast amounts of data, thereby improving its predictive accuracy over time. In the context of non-invasive vital sign signal monitoring, deep learning models can be utilized to automatically identify and analyze vital sign signals such as heart rate, blood pressure, and oxygen saturation. This automated process not only improves the efficiency of monitoring but also reduces the likelihood of human error, thereby enhancing the overall accuracy of the monitoring process.

Fiber optic sensors, on the other hand, are a novel sensor technology that detects physical parameters by measuring changes in light propagation through optical fibers [4]. These sensors can be used for contactless measurement of vital sign signals in non-invasive monitoring, thereby significantly improving patient comfort and satisfaction [5]. The use of fiber optic sensors eliminates the need for direct contact with the patient, thereby reducing discomfort and allowing for greater patient mobility during monitoring.

Present studies in this domain mainly concentrate on improving the precision and instantaneous functionality of models based on deep learning for monitoring vital signs non-invasively through the use of fiber optic sensors. Researchers are also striving to minimize the impact of environmental noise on the monitoring process, thereby improving the reliability of the results.

In addition, there is a growing interest in exploring the application of these models in various scenarios such as telemedicine and home healthcare. The ability to accurately monitor vital signs remotely could significantly enhance the accessibility and convenience of healthcare services, particularly for patients with mobility issues or those living in remote areas.

Despite the significant potential of deep learning-based non-invasive vital sign signal monitoring models for fiber optic sensors, the field still faces numerous challenges. These include improving the accuracy and reliability of the models, addressing data privacy and security concerns, and ensuring the models are robust enough to handle the variability and complexity of real-world data.

Furthermore, the successful implementation of these models in a clinical setting requires the development of appropriate regulatory frameworks and guidelines to ensure patient safety and data integrity. It also necessitates the training of healthcare professionals to effectively use and interpret the results generated by these models.

Building on our previous research, we have further streamlined the physical structure of the system. This simplification process involved a careful analysis of the system’s components and their functions. We identified areas where the system could be made more efficient without compromising its performance. This resulted in a more compact that is easier to maintain and operate. Moreover, we have enhanced the data preprocessing operation from its original form. We recognized that the initial process was prone to generating unnecessary errors that could compromise the accuracy of our results. To address this, we implemented more rigorous data validation techniques and error-checking protocols. These improvements have significantly reduced the occurrence of unnecessary errors, leading to more reliable and accurate data processing.

In conclusion, the integration of deep learning and fiber optic sensor technology in non-invasive vital sign signal monitoring represents a promising avenue for future research. While the field still faces numerous challenges, the potential benefits in terms of improved accuracy, efficiency, and patient comfort make it a worthwhile endeavor. With continued research and development, this innovative approach could significantly transform the landscape of healthcare monitoring and diagnosis.

In comparison to similar studies at this stage, we employ the DEMA model, a technique that accurately predicts trends, especially in volatile data. This is paired with non-invasive contact detection, a method that collects crucial data without causing discomfort to patients. The combination of these two techniques enhances efficiency and accuracy, leading to more precise detection results. Consequently, this results in a significant reduction in misdiagnosis rates in the medical field, as the accurate diagnosis is crucial for effective treatment.

## 2. Methodology

### 2.1. RNN and LSTM in Time-Series Data

For the past few years, RNNs have emerged as a mighty tool in time-series data processing, achieving remarkable results that have significantly advanced the field. RNNs, a category of artificial neural networks specifically engineered to identify patterns in sequential data like text, genomes, handwriting, or spoken language, have demonstrated significant effectiveness in handling such data types [6]. Unlike traditional methods such as Fast Fourier Transform (FFT) and Wavelet Transform (WT), which necessitate handcrafted feature extraction and may frequently overlook complex patterns within the data, deep learning techniques such as RNNs have the capability to autonomously extract valuable features from unprocessed signals [7]. This is a significant advantage as it eliminates the need for manual feature engineering, which is often time consuming and requires expert knowledge. Moreover, RNNs are capable of reflecting internal data properties, which means they can capture the inherent characteristics and structures within the data [8]. The significance is especially pronounced in time-series data, wherein the temporal dynamics and dependencies exert a pivotal influence. By capturing these dynamics, RNNs can provide a more accurate and nuanced understanding of the data. Furthermore, the use of RNNs can significantly improve algorithm efficiency. Traditional algorithms often struggle with large-scale data and high-dimensional inputs, but RNNs, with their deep learning capabilities, can handle such complex data with relative ease. They can process large amounts of data in a relatively short time, making them highly efficient and scalable. In conclusion, the advent of RNNs has had a profound impact on time-series data processing [9]. The capacity to extract features from unprocessed signals, mirror intrinsic data characteristics, and augment algorithmic efficiency has amplified the precision and effectiveness of data processing and inaugurated novel avenues for examining and elucidating time-series data.

**Figure 1:**
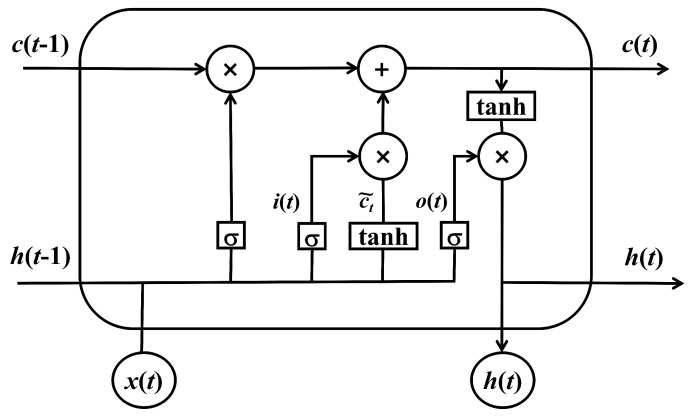
Hidden layer of LSTM.

LSTM represents a sophisticated modification of RNNs, explicitly designed to tackle the challenge of long-term dependencies in sequential data manipulation. Unlike traditional RNNs, LSTM integrates gate functions and memory components, thus augmenting its ability to apprehend and preserve long-term dependencies. The refinement of model parameters in LSTM is accomplished via the backpropagation algorithm, which is oriented towards the reduction in prediction discrepancies. The LSTM architecture is frequently executed using a specialized component in neural networks called the LSTM cell, which comprises a memory unit and three regulatory units: the input gate, forget gate, and output gate, as shown in Figure 1. These regulatory units empower the LSTM to manage the information’s flow and retention, considering both the present input and the antecedent hidden state [3,4]. This distinctive configuration permits the LSTM to adeptly manage long-range dependencies and circumvent problems such as vanishing and exploding gradients, which are prevalent in conventional RNNs. In the realm of time-series data processing, particularly for tasks necessitating extended contextual information, LSTM has demonstrated its superior capabilities. This includes fields like natural language processing and speech recognition, where LSTM’s ability to consider long-term dependencies proves particularly beneficial [10]. The equations for the forward progression of an LSTM cell equipped with a forget gate, with the variables included, are as follows:

(1) $$f_{t}=\sigma_{g}\left(W_{f}x_{t}+U_{f}h_{t-1}+b_{f}\right)$$

(2) $$i_{t}=\sigma_{g}\left(W_{i}x_{t}+U_{i}h_{t-1}+b_{i}\right)$$

(3) $$o_{t}=\sigma_{g}\left(W_{o}x_{t}+U_{o}h_{t-1}+b_{o}\right)$$

(4) $${\tilde{c}}_{t}=\sigma_{c}\left(W_{c}x_{t}+U_{c}h_{t-1}+b_{c}\right)$$

(5) $$c_{t}=f_{t}⊙c_{t-1}+i_{t}⊙{\tilde{c}}_{t}$$

(6) $$h_{t}=o_{t}⊙\sigma_{h}\left(c_{t}\right)$$

The initial values are denoted by $c_{0}=0$ and $h_{0}=0$ the operator ⊙ signifies the Hadamard product (also known as element-wise product).

The subscript *t* is used to index the time step.

$W\in R^{\left(h\times d\right)}$, $U\in R^{\left(h\times h\right)}$ and $b\in R^{h}$ are the weight matrices and bias vector parameters that need to be optimized during the training process.

The superscripts are used to denote the number of input features and the number of hidden units, respectively [11].

$f_{t}\in {\left(0,1\right)}^{h}$ is the activation vector of the forget gate.

$x_{t}\in R^{d}$ represents the input vector that is fed into the LSTM unit.

$i_{t}\in {\left(0,1\right)}^{h}$ is the activation vector of the input/update gate.

$o_{t}\in {\left(0,1\right)}^{h}$ is output gate’s activation vector.

${\tilde{c}}_{t}\in {\left(-1,1\right)}^{h}$ is the activation vector for cell input.

$c_{t}\in R^{h}$ is the vector for cell state.

$h_{t}\in {\left(-1,1\right)}^{h}$ is hidden state vector also known as output vector of the LSTM unit.

### 2.2. EMD and LSTM Integration in Signal Processing

EMD stands as an advanced method in signal processing that breaks down non-stationary signals into multiple Intrinsic Mode Functions (IMFs), shedding light on the signal’s local characteristics [5,12]. The defining feature of the EMD method is its flexibility, allowing it to produce suitable IMFs based on the unique attributes of the signal. This flexibility makes EMD particularly beneficial for analyzing signals characterized by non-linearity and non-stationarity, such as various physiological signals like heart and respiratory rates. Nonetheless, the EMD method is not without its flaws, with mode mixing being a significant issue. Mode mixing occurs when oscillation modes of similar types are present at different amplitude levels, or when several distinct sub-modes are found within a single mode.

In comparison, while both LSTM and EMD have their unique strengths in handling specific types of data and tasks, they also have their respective limitations. LSTM excels in processing time-series data and managing long-term dependencies, but it requires careful tuning of parameters and can be computationally intensive. On the other hand, EMD is adept at decomposing non-stationary signals to reveal local characteristics, but it suffers from the issue of mode mixing. Therefore, the choice between LSTM and EMD would depend on the specific requirements and constraints of the task in question.

The integration of EMD and LSTM presents a novel approach to signal processing and sequential data analysis. This amalgamation leverages the strengths of both techniques, thereby enhancing the overall performance and efficiency of the system. Given its capability to break down non-stationary signals into IMFs, EMD efficiently uncovers the local features of the signal. This disintegration procedure can function as an initial processing stage, transmuting the raw signal into a collection of IMFs that encapsulate the fundamental characteristics of the signal [13]. These IMFs can then be used as inputs to the LSTM model. On the other side, LSTM excels in processing time-series data and managing long-term dependencies. By feeding the IMFs generated by EMD into LSTM, the model has the ability to learn and capture the temporal dynamics, and dependencies from these IMFs. This can potentially lead to more accurate and robust predictions, as the model can now consider both the local features of the signal (captured by EMD) and the temporal dependencies among these features (captured by LSTM) [14].

The integration of EMD and LSTM is particularly significant in applications where both local features and temporal dependencies are crucial. For instance, in physiological signal analysis, the local features of the signal (such as the amplitude and frequency of heartbeats or breaths) and the temporal dependencies among these features (such as the regularity and rhythm of heartbeats or breaths) are both important for accurate diagnosis and prediction. By combining EMD and LSTM, we can potentially achieve more accurate and robust analysis and prediction in these applications.

### 2.3. Design of Optical Fiber Monitoring

**Figure 2:**
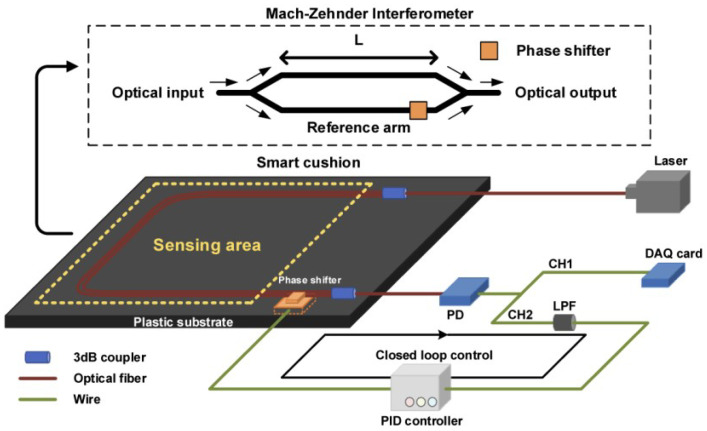
The proposed optical fiber sensor system [15].

**Figure 3:**
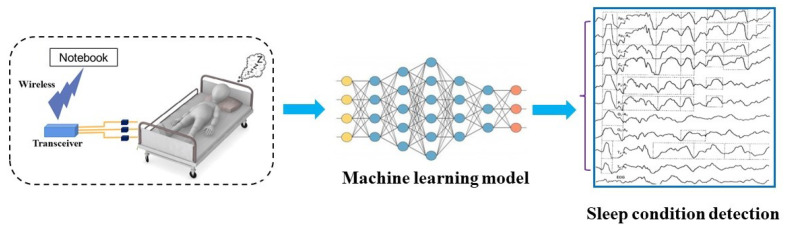
The overall process of human vital sign signal monitoring.

In this study, we introduce an innovative optical fiber sensor designed to function as a non-invasive system for the monitoring of vital signs, as shown in Figure 2 and Figure 3. This monitoring system comprises several pivotal elements, encompassing a BCG monitor functioning based on an MZI, a PID controller, and a phase modulator.

The BCG monitor, serving as the central component of the surveillance system, is built upon the principles of the MZI. The MZI is an apparatus to ascertain the relative phase shift alterations between two collimated beams originating from coherent light sources. In this scenario, it is deployed to oversee the mechanical functioning of the heart, as depicted by the BCG.

**Figure 4:**
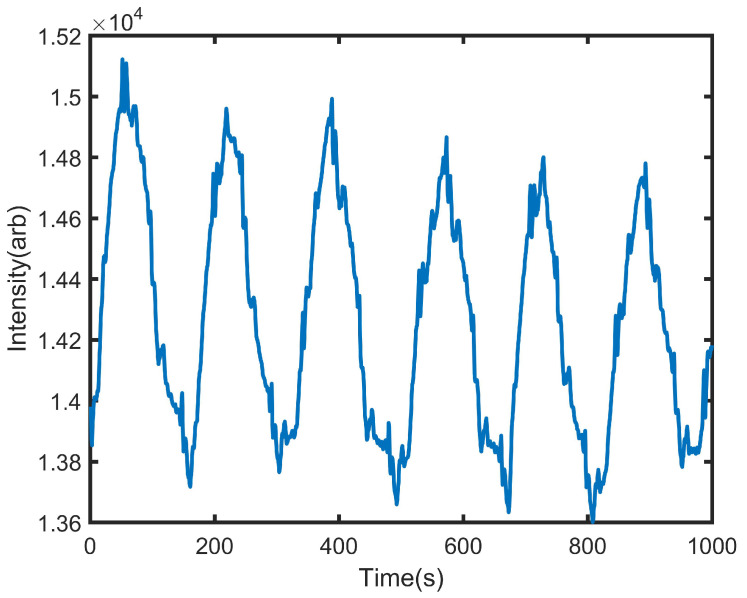
Raw signal acquired by the proposed optical fiber sensor.

The monitoring system also integrates a phase shifter, a distributed feedback (DFB) laser, and a low-speed photodetector (PD). The phase shifter is an apparatus that modifies the phase of a light wave traversing the optical fiber, while the DFB laser is employed to produce the coherent light source requisite for the MZI. Conversely, the PD is tasked with transforming the BCG signal into an electrical signal amenable to further processing. The envisioned system for monitoring, integrated into a cushion, is built around a Mach-Zehnder Interferometer (MZI)-based Ballistocardiogram (BCG) monitor, a phase shifter, and a proportional-integral-derivative (PID) controller. Structurally, the cushion is composed of four layers: the top material layer, a pressure detection layer, a sponge layer, and the bottom material layer, arranged from top to bottom. The MZI in the optical fiber features two 3dB couplers that serve both as optical splitters and couplers, creating interference. Both the MZI and the phase shifter are mounted on a plastic base, which is then encased in a cushion design, facilitating non-invasive BCG monitoring. The MZI’s arms, the sensing and reference arms, are aligned in parallel. Positioned outside the detection zone, the phase shifter, controlled by a PID controller, ensures the interferometer system remains in quadrature. The interferometer’s arms are approximately 40 cm in length with a 5 mm difference between them, shaped into a semicircle side by side without overlapping, and encased on a plastic base within the cushion. Signal fading, a shift in the bias point leading to sensitivity alterations and potential BCG signal distortion, is a noted challenge. To tackle this challenge, a variety of phase modulation strategies are utilized, encompassing both active and passive homodyne techniques. Our technique adopts the active homodyne method, incorporating a compact moving-coil transducer to act as the phase shifter and preserve system quadrature, which allows for its effortless incorporation into the intelligent cushion design. The moving-coil transducer, serving as the phase shifter in our BCG detection system, has dimensions of 18 (Length) × 12 (Width) × 3 (Height) mm. This facilitates its straightforward integration into the cushion-based BCG monitoring device, offering an advantage over the more cumbersome piezoelectric transducer-based (PZT) phase modulation techniques. Additionally, this approach avoids extra bending loss since the optical fiber is directly connected to the transducer instead of being wound around it. As illustrated in Figure 4, the specific area designated for capturing BCG signals from individuals in a seated position is highlighted by a yellow dashed rectangle. The configuration employs a DFB laser as the illumination source and a slow photodetector (PD) to transform variations in the optical signal related to BCG into electrical signals. The output from the PD is divided into two pathways: Channel 1 (CH1) conveys the raw data, whereas Channel 2 (CH2) directs the data through a low-pass filter (LPF), which then inputs into the PID controller. This controller compensates for phase fluctuations, ensuring the system remains at the quadrature point through adjustments made by the phase shifter.

The MZI is assembled with 23 dB couplers that serve both as an optical splitter and coupler, and it is affixed to a plastic base. This setup allows for its encapsulation as a smart cushion, enhancing its versatility and user-friendliness. The MZI along with the phase shifter are strategically placed outside the detection zone and are aligned in parallel to guarantee peak performance. This careful positioning prevents potential interference and promotes efficient light signal transfer, thereby guaranteeing the device’s peak performance.

The electrical signal produced by the PD is partitioned into two channels, CH1 and CH2. CH1 encompasses the unprocessed data, whereas CH2 incorporates the data that have undergone processing via a low-pass filter (LPF) [15]. The LPF serves to eliminate high-frequency noise from the signal, thereby improving the accuracy of the data. This processed data are then used as the input of the PID controller, a feedback mechanism of the control loop extensively utilized in industrial control frameworks. The PID controller adjusts the control inputs to the system based on the error between the desired and actual output, thereby ensuring the stability and accuracy of the monitoring system.

### 2.4. EMD Algorithm

The EMD algorithm was introduced by Huang and colleagues in the 1990s and is a data decomposition method that breaks down non-stationary signals into IMFs [16]. These IMFs are more straightforward oscillatory functions that shed light on the signal’s composition and dynamics. The breakdown procedure adheres to a pair of criteria: (1) the aggregate count of extrema and zero-crossings should be identical or have a discrepancy of no more than one, which guarantees a harmonious oscillatory nature, and (2) the average value of the upper and lower envelopes, which are determined by the local peaks and troughs, ought to be null, signifying symmetry around the horizontal axis. Adherence to these conditions is vital as it ensures each IMF has a mean value of zero, which is essential for accurately determining the signal’s instantaneous frequency [11].

The EMD decomposition is realized as follows:

(1) The extreme points of the original time series data $x_{t}$ are fitted using cubic spline interpolation to obtain the upper and lower envelopes $u_{t}$ and $d_{t}$.

(2) Compute the average sequence of the superior and inferior envelopes $m_{1}\left(t\right)$:

(7) $$m_{1}\left(t\right)=\frac{u_{t}+d_{t}}{2}$$

(3) The raw time series data $x_{t}$ is differenced from the envelope mean sequence $m_{1}\left(t\right)$ to obtain the intermediate time series $h_{1}\left(t\right)$:

(8) $$h_{1}\left(t\right)=x_{t}-m_{1}\left(t\right)$$

(4) Determine whether $h_{1}\left(t\right)$ satisfies the IMF condition, if so, $h_{1}\left(t\right)$ is the IMF part from the original data. If not, iterate Steps (1)–(4) until the new provisional time series $h_{1}k\left(t\right)$ satisfies the IMF condition and the standard deviation SD is less than the set value. After that, $h_{1}k\left(t\right)$ is used as the first IMF component of the original data, denoted as $c_{1}\left(t\right)$:

(9) $$c_{1}\left(t\right)=h_{1k}\left(t\right)$$

(10) $$SD=\sum_{t=0}^{T}\frac{|h_{1(k-1)}\left(t\right)-h_{1k}{\left(t\right)|}^{2}}{h_{1(k-1)}^{2}\left(t\right)}$$

(5) The original timing data $x_{t}$ is compared with $c_{1}\left(t\right)$ to obtain the remaining sequence $r_{1}\left(t\right)$:

(11) $$r_{1}\left(t\right)=x_{t}-c_{1}\left(t\right)$$

(6) Iterate $r_{1}\left(t\right)$ by the original data and iterate Steps (1)–(5) until the remaining sequence $r_{n}\left(t\right)$ is a unidirectional function or a fixed value, procure the corresponding IMF elements $c_{1}\left(t\right)$, $c_{2}\left(t\right)$, …, $c_{n}\left(t\right)$, and end the decomposition [17].

The original timing data are decomposed by EMD to obtain n IMF elements $c_{1}\left(t\right)$, $c_{2}\left(t\right)$, …, $c_{n}\left(t\right)$, and the residue sequence $r_{n}\left(t\right)$:

(12) $$x\left(t\right)=\sum_{i=1}^{n}c_{1}\left(t\right)+r_{n}\left(t\right)$$

### 2.5. EEMD Algorithm

In the process of employing EMD for signal decomposition, a phenomenon known as modal aliasing is encountered. This pertains to the phenomenon where signals with multiple time-scale attributes are encapsulated within a single IMF component, or the scattering of the time-scale characteristics of one IMF component into additional IMF components. During the EMD decomposition process, each IMF component is susceptible to modal aliasing, which is subsequently transferred to the following IMF components as the decomposition progresses, leading to signal distortion. The root cause of this modal aliasing issue can be traced back to the frequent shifts and alterations of the local extreme value points during the EMD decomposition process. In real-world scenarios, the data collected are invariably tainted with noise, making the noise-infected signal more susceptible to modal aliasing. To address the issue of mode mixing associated with EMD, Handrin and his team proposed a refined EMD approach based on noise-assisted analysis, known as Ensemble Empirical Mode Decomposition (EEMD). This technique entails the decomposition of signals post the addition of white noise through EMD [18]. The fundamental concept behind EEMD is to perform several empirical mode decompositions with the inclusion of Gaussian white noise, capitalizing on the statistical characteristic of the noise’s frequency-uniform distribution. By adding different instances of white noise with the same amplitude to modify the characteristics of the signal’s extremities, the IMFs obtained from various EMD processes are averaged. This averaging process aims to cancel out the effect of the added white noise, effectively reducing the likelihood of mode mixing. The EEMD algorithm emerges as an effective tool for analyzing and processing non-linear and non-stationary signals, offering a solution to the mode mixing challenge in signal decomposition. However, it is not without its drawbacks, including (1) the presence of residual white noise during decomposition, and (2) the reliance on experiential judgment for selecting effective IMFs [19,20].

The EMD decomposition is realized as follows: add the noise $\omega_{n}\left(t\right)$, n=1,2,…,N to the original timing data X(t) to obtain the timing data $X_{n}\left(t\right)$ to be decomposed:

(13) $$X_{n}\left(t\right)=X\left(t\right)+\omega_{n}\left(t\right)$$

$X_{n}\left(t\right)$ is decomposed by EMD to obtain the corresponding IMF component $c_{i},n_{t},i=1,2,\ldots ,m$. $r_{m,n}\left(t\right)$ as the remaining sequence:

(14) $$X_{n}\left(t\right)=\sum_{i=1}^{m}c_{1,n}\left(t\right)+r_{m,n}\left(t\right)$$

Iterate the above two steps, incorporating fresh white noise at each instance, for obtaining the set of N IMF components and the remaining sequences [21]. The last step is obtained by averaging the above N sets of IMF elements as well as the set of remaining sequences:

(15) $$c_{n}\left(t\right)=\frac{1}{n}\sum_{i=1}^{N}c_{in}\left(t\right)$$

(16) $$r_{m}\left(t\right)=\frac{1}{N}\sum_{n=1}^{N}r_{m,n}\left(t\right)$$

### 2.6. DEMA Algorithm

The computational procedure required to attain the intended signal decomposition outcomes utilizing the EEMD algorithm can be time consuming. Furthermore, in instances where the data are supplemented with inadequate white noise, the signal reconstructed through EEMD decomposition will likely retain residual auxiliary signals. To address the mode mixing problem found in the EMD algorithm, the EEMD and Complete EEMD decomposition techniques reduce the mode mixing in EMD decomposition by adding both positive and negative Gaussian white noise to the set of signals being decomposed. However, these techniques often result in some remaining white noise within the extracted IMFs, which can affect further signal analysis and processing. To overcome these drawbacks, Torres et al. proposed a more sophisticated algorithm: the DEMA. By incorporating a noise-assisted mechanism and an adaptive noise standard deviation, the DEMA algorithm improves the resilience and precision of EMD when dealing with non-stationary signals [22,23]. Its use has been widely recognized in the fields of signal processing and vibration analysis, demonstrating enhanced performance over the conventional EMD in certain cases.

## 3. Experiment and Discussion

### 3.1. Data Resources and Processing

Initially, an adaptive heart rate calculation approach based on Bayesian probability is used to collect ECG data from the heartbeat interval sequence [15]. Each sequence of five-minute heartbeat intervals is termed a sample. For classification, a proposed machine learning model is applied following preprocessing, feature extraction, and feature dimensionality reduction. The categorization’s outcomes serve to validate the algorithm’s viability.

The adaptive heart rate is determined using Bayesian probability to determine the order of heartbeat intervals. Ectopic beats can cause errors in the time and frequency domain analysis of HRV, which is why the interval sequence needs to be preprocessed. Upper and lower bounds are specifically defined to detect and rectify data that are more than 1.3 times the average value of the R-R interval and falls below 0.7 times the average value of the R-R interval [24]. The outlier’s adjustment value is determined by the correction technique by squaring the median value of the thirty data points surrounding it. Once the data are processed, the interval values are maintained within the standard threshold range [25].

After the heartbeat interval sequence has been preprocessed, data points that fall within a five-minute window are considered a sample, and the characteristics of the sample data in the frequency and temporal domains are retrieved. To reduce the total forward and backward prediction error power, the order of the autoregressive (AR) model is set at 16 using mathematical statistics techniques [26]. To estimate the power spectrum and get the AR coefficient, Levenson Durbin recursion is used. The next step is to apply principal component analysis to reduce the dimensionality of the feature matrix [27]. This allows for the extraction of features from the samples and increases operational efficiency by using fewer feature vectors to represent the original data.

The process of data processing by the DEMA model typically involves the following steps: (1) Data Preparation: the first step is to prepare the input data, which may involve tasks such as data cleaning, normalization, and feature engineering to ensure that the data are suitable for training and prediction by the DEMA model. (2) Forward Propagation: the input data are passed through the DEMA’s layers, where they undergo weighted summation and activation function processing, propagating through each layer until they reach the output layer. At each layer, the DEMA model computes and outputs a new set of features. (3) Loss Computation: the predicted output from the output layer is compared with the actual labels, and a loss function is calculated to measure the difference between the predicted values and the actual values. (4) Backward Propagation: using the backpropagation algorithm, the neural network adjusts the weights and biases of each layer based on the gradient of the loss function, aiming to minimize the loss. This process uses gradient descent to update the model parameters, allowing the DEMA model to better fit the data. (5) Iterative Training: the above steps are repeated multiple times until the model converges or reaches a predefined stopping condition. During training, a validation set can be used to monitor the DEMA’s performance and make hyperparameter adjustments. (6) Model Evaluation: once training is complete, the DEMA’s performance is evaluated using test data, including metrics such as RMSE, MAE, and R².

### 3.2. Experimental Preparation and Results

In our study, to assess the proposed model’s performance and compare it with alternative models, we collected 3 hours of ballistocardiography (BCG) data from 20 participants, dividing this data into an 80% training set and a 20% test set. The model’s implementation was carried out using the PyTorch framework and Python programming language, with the ADAM optimizer, a batch size of 1, and a learning rate of 0.0001. Additionally, we employed 10-fold cross-validation to further divide the dataset for training, validation, and testing purposes. This method allowed for continuous refinement of the model through repeated tuning and training, leading to a consistent decrease in the error rate and thus improved accuracy. The validation subset played a crucial role in fine-tuning the model’s parameters, enhancing its ability to generalize and make accurate predictions on new data. To prevent overfitting, a dropout strategy was also implemented, ensuring the model’s robustness and reliability.

Regarding the analysis of BCG signals, the precise gathering and interpretation of HRV signals are imperative for assessing the cardiorespiratory condition. HRV analysis quantifies the fluctuations in time intervals between successive heartbeats and examines a range of indicators, encompassing both time-domain and frequency-domain measures. These indicators offer valuable perspectives on the equilibrium of the autonomic nervous system (ANS), levels of stress, recuperation rates, and potential indicators for various medical conditions, establishing HRV as a non-invasive and potent instrument for gauging ANS function and overall well-being.

Heart rate (HR) and respiratory rate (RR) were chosen as our study’s primary indicators for evaluation. To quantify the accuracy and performance of the assessments, the study utilized root mean square error (RMSE), mean absolute error (MAE), and the coefficient of determination (R²) as the evaluation metrics. RMSE represents the square root of the average of the squared differences between the predicted and actual values, serving as an index of the variations between observed and true values. MAE calculates the average of the absolute differences between predictions and actual values, providing a measure of the average magnitude of errors in the predictions. R², also known as the coefficient of determination, quantifies the fit quality of the regression model by indicating the proportion of variance in the dependent variable that is predictable from the independent variables in the model. We refer to the support vector regression (SVR), backpropagation (BP), and long short-term memory (LSTM) models, and counted the data derived from 10 volunteers through four different models. In Table 1, when the Dynamic Exponential Moving Average (DEMA) model performs best, the data of the individuals involved are emphasized. By statistically analyzing the data, we can clearly observe that the DEMA model still performs excellently even under different metrics.

(17) $$RMSE=\sqrt{\frac{1}{m}\sum_{i=1}^{m}{\left(y_{i}-\hat{y_{i}}\right)}^{2}}$$

(18) $$MAE=\frac{1}{n}\sum_{i=1}^{n}\left|\hat{y_{i}}-y_{i}\right|$$

(19) $$R^{2}=1-\frac{\sum_{i}{\left(\hat{y_{i}}-y_{i}\right)}^{2}}{\sum_{i}{\left(\bar{y_{i}}-y_{i}\right)}^{2}}$$

**Figure 5:**
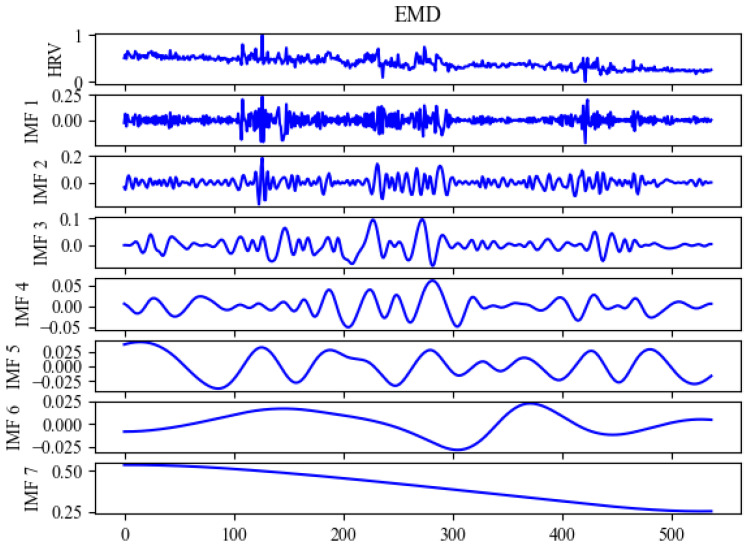
Signal modal decomposition diagram by EMD per second.

**Figure 6:**
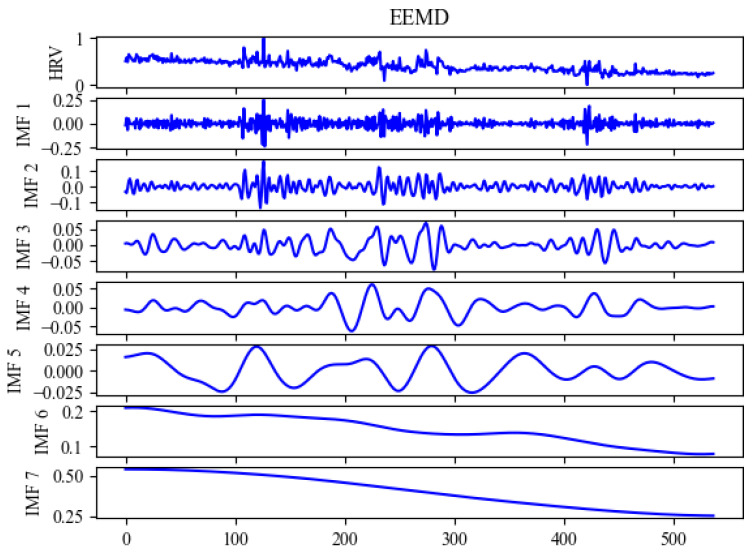
Signal modal decomposition diagram by EEMD per second.

**Figure 7:**
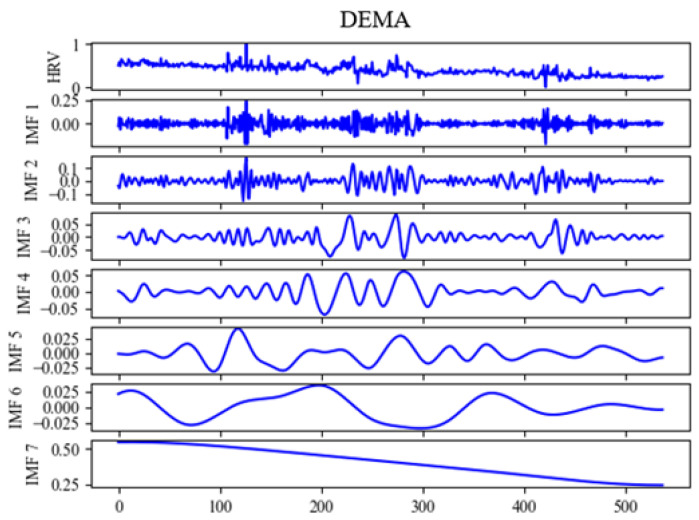
Signal modal decomposition diagram by DEMA per second.

**Figure 8:**
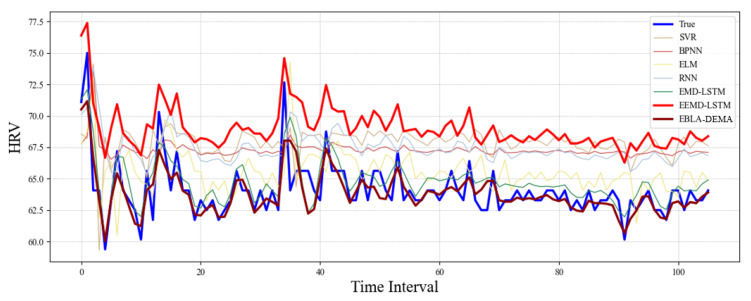
HRV measurement results by all the models per second.

In our research, we utilized SVR, ELM, BPNN, and RNNs as benchmark models for evaluating our proposed model, as shown in Table 2. The models were first subjected to four preliminary successive experiments to establish a base benchmark. Subsequent experiments were carried out using EMD as shown in Figure 5, EEMD as shown in Figure 6, and DEMA as shown in Figure 7, with the outcomes visually depicted in the associated figure. Analysis of these results revealed that the benchmark models suffered from underfitting in comparison to the actual data. This underperformance was linked to inadequate feature extraction, leading to discrepancies in curve trends and notably elevated HRV measurements. On the other hand, EEMD showed improved alignment with the actual data curves but still presented elevated HRV figures, suggesting insufficient extraction of trends. For better visual representation, DEMA is referred to as EBLA in Figure 8. Further scrutiny revealed that DEMA’s performance, in terms of both curve alignment and HRV metrics, was significantly superior to that of the other models. This suggests that DEMA was more effective in capturing the underlying trends and patterns in the data.

### 3.3. Summary

In our study, we chose four reference models for comparison with the model we designed. These models include SVR, ELM, BPNN, and RNNs. These models were selected due to their proven effectiveness in similar studies and their diverse methodologies, which provide a comprehensive comparison of our designed model.

The initial four experiment phases were carried out one after the other, with each phase utilizing one among the four benchmark models. This method enabled the independent assessment of each model’s effectiveness, allowing for the identification of their respective advantages and disadvantages. The results from these initial rounds of experiments provided valuable insights that guided the subsequent stages of our study.

Following the initial rounds, we conducted further experiments using EMD, EEMD, and DEMA. These methodologies were selected owing to their capacity to disintegrate a signal into a limited number of intrinsic mode functions, which can be advantageous for feature extraction.

The results of our experiments are illustrated in the accompanying figure. Upon analysis, it becomes clear that the reference models did not deliver as high a performance as expected. They demonstrated underperformance relative to the ground truth, a situation we ascribe to inadequate feature extraction. Consequently, this led to trend lines in the curves that strayed from the anticipated trajectory and markedly elevated HRV measurements.

In contrast, EEMD demonstrated a better fit to the ground truth curve. However, it also produced substantially higher HRV values, which we believe is due to inadequate trend extraction. This suggests that while EEMD can capture the overall shape of the curve, it struggles to capture the finer details accurately.

In contrast to the earlier research by Wang and colleagues [24], (1) sensors that rely on loss are prone to damage during operation and do not match the sensitivity levels of optical fiber interferometer sensors. (2) The expense associated with grating-based sensors is considerable, posing challenges for their practical deployment. (3) Sensors that utilize electrical principles for dual sensing exhibit inadequate responsiveness to faint vibrational signals and struggle to yield high-fidelity signals amidst complex settings. The optical fiber sensor introduced in this investigation effectively mitigates ambient noise and enhances the integrity of the acquired signal. The monitoring framework presented here is straightforward, economical, and non-intrusive. The fast Fourier transform (FFT) and wavelet transform (WT), employed in prior studies, are conventional techniques. These established techniques depend on predetermined feature extraction methods to isolate pertinent data, which invariably leads to some degree of information loss. In this paper, the proposed DEMA model is more robust to noise and outliers in the input data compared to traditional algorithms. It can also lead to more accurate and robust feature extraction compared to traditional algorithms.

For clarity in our discussion, we have denoted DEMA as EBLA. Upon analysis, it is evident that EBLA outperformed the other models in both curve trends and HRV values. This suggests that EBLA is capable of more accurate feature and trend extraction, making it a promising tool for future studies.

## 4. Conclusions and Future Works

### 4.1. Research Summary

In this research paper, we have introduced a groundbreaking non-contact monitoring system that utilizes a micro-bend fiber sensor. This innovative system represents a significant departure from traditional monitoring systems that rely on FFT and WT algorithms. The newly developed model is specifically designed to significantly reduce errors, thereby enhancing the overall efficiency and reliability of the system.

One of the primary challenges that this model addresses is the pattern mixing problem, a common issue in pure EMD algorithms. By integrating LSTM with the DEMA model, we have successfully mitigated this problem. The suggested model has shown considerable enhancements across different performance indicators, such as accuracy, precision, and recall. These improvements are not merely incremental but are substantial enough to enhance the accuracy of medical diagnostic measurements significantly.

The implications of this model are far-reaching and transformative. It holds the potential to revolutionize the way medical diagnostic measurements are conducted, making them more accurate, reliable, and efficient. This could lead to a paradigm shift in healthcare delivery, with non-contact monitoring becoming the norm rather than the exception.

Moreover, the enhanced accuracy and reliability of diagnostic measurements could lead to better patient outcomes. By providing clinicians with more accurate data, this model could facilitate more precise diagnoses, more effective treatment plans, and more proactive healthcare management. This model could also improve healthcare delivery by making it more efficient and cost-effective. By reducing errors and improving accuracy, this model could minimize the need for repeat tests and unnecessary interventions, thereby saving time and resources.

In conclusion, the novel non-contact monitoring system presented in this paper represents a significant advancement in the field of medical diagnostics. With its potential to enhance accuracy, improve patient outcomes, and streamline healthcare delivery, this model could pave the way for a new era of non-contact monitoring in healthcare.

### 4.2. Future Works

As we look towards the future, we have identified several ambitious goals that we aim to achieve. Our immediate task is to broaden the application of our current achievement into wider domains. We are confident that the principles and techniques utilized in this model can be extrapolated to other areas, thereby expanding its utility and impact. This could include applications in remote patient monitoring, home healthcare, and even fitness and wellness tracking. The potential to revolutionize these areas with our non-contact monitoring system is immense and we are excited to explore these possibilities.

Designing simplified experiments or configurations for this system represents another difficult but essential goal on our future research agenda. We aim to make the system more accessible and user-friendly, increasing its adoption in various settings. This involves refining the user interface, simplifying the setup process, and providing clear instructions and support for users. By doing so, we hope to make our non-contact monitoring system a practical tool for everyday use, whether in a clinical setting or at home [28].

Additionally, we are enthusiastic about pursuing detailed conversations and possible collaborative efforts with other fields, including EEG and the study of human ergonomics. We believe that such interdisciplinary collaborations can lead to innovative solutions and breakthroughs. For instance, integrating EEG data could allow our system to monitor neurological activity, providing valuable insights into cognitive health. Similarly, insights from human ergonomics could help us design a more comfortable and user-friendly device, thereby enhancing user experience and adherence.

In addition to these goals, we also aim to address potential challenges and limitations of our system. This includes improving the accuracy and reliability of our system, ensuring data privacy and security, and making our system robust enough to handle the variability and complexity of real-world data. We also plan to conduct rigorous validation studies to ensure the clinical relevance and effectiveness of our system.

Lastly, we are committed to fostering a culture of continuous learning and innovation. We plan to stay abreast of the latest research and technological advancements in our field and to incorporate these into our system wherever possible. We believe that this commitment to innovation and excellence will enable us to continually improve our system and to stay at the forefront of the field of non-contact monitoring systems.

In conclusion, we are excited about the future of our non-contact monitoring system. We believe that with continued research and development, our system has the potential to significantly transform the landscape of health monitoring and to bring about significant benefits for users worldwide.

## Figures and Tables

**Table 1:** Summary of the proposed model.

Layer (Type)	Output Shape
Conv1	(None, 6, 1, 56, 32)
LeakyRelu1	(None, 6, 1, 56, 32)
Maxpooling1	(None, 6, 1, 28, 32)
Dropout1	(None, 6, 1, 28, 32)
Conv2	(None, 6, 1, 28, 64)
LeakyRelu2	(None, 6, 1, 28, 64)
Maxpooling2	(None, 6, 1, 14, 64)
Dropout2	(None, 6, 1, 14, 64)
Fullyconnect1	(None, 6, 512)
LeakyRelu3	(None, 6, 512)
LSTM1	(None, 6, 512)
LSTM2	(None, 222)
Fullyconnect2	(None, 222)
Softmax	(None, 222)

**Table 2:** Experimental results based on different methods.

		HR				RR			
Metric	Subject	SVR	BP	LSTM	DEMA	SVR	BP	LSTM	DEMA
	#1	2.4928	2.1393	2.7355	2.7463	1.4045	1.2844	0.9645	0.6455
	#2	6.1216	2.1342	0.9866	0.1213	2.1301	1.3203	1.7404	0.5321
	#3	2.7937	1.5464	0.5027	0.4943	2.4852	1.4765	0.7035	0.5213
	#4	3.2752	4.4643	3.6292	3.4753	0.5770	1.7979	0.6069	1.4153
RMSE	#5	1.1852	2.7873	1.0835	0.8944	1.7928	1.9267	1.0789	1.0783
	#6	5.0871	5.6618	5.9866	4.3185	0.7724	1.6106	0.7721	0.6306
	#7	0.7704	1.2019	0.6527	0.4413	0.7689	1.3055	1.0115	1.9046
	#8	3.6025	6.6618	6.1989	6.2372	0.2149	0.9005	0.1716	0.0178
	#9	1.6989	2.4858	2.0137	1.0418	0.2701	0.4252	0.1776	0.1773
	#10	0.8569	1.1270	0.9934	0.0981	1.4391	0.9743	1.4435	0.2236
	#1	1.2679	3.2425	2.3323	2.3734	3.9909	3.8487	5.1445	4.5031
	#2	5.0499	4.5413	4.7483	3.0294	4.5664	4.0096	5.6250	3.3313
	#3	2.1315	2.0357	2.5423	1.2899	4.8342	4.9337	4.0027	3.2391
	#4	2.6969	3.3541	2.7902	2.6000	4.2679	4.8385	4.3408	4.2623
MAE	#5	5.9401	6.8456	5.8942	5.7944	6.6311	8.1211	6.8522	6.5737
	#6	3.8128	2.8181	3.1676	2.0548	6.3954	7.0754	6.3953	6.3564
	#7	6.5097	6.7780	6.6013	6.2018	7.5440	7.6954	7.1882	6.1065
	#8	5.5451	6.8181	5.6584	5.5975	0.0662	0.8513	0.0361	0.0337
	#9	3.3111	3.0900	3.6194	2.6336	0.1630	0.1175	0.0513	0.0483
	#10	5.6370	4.9305	5.8267	5.0192	1.0615	1.2426	0.9195	0.0692
	#1	0.9386	0.6920	0.9869	0.9687	0.9416	0.8213	0.6457	0.7991
	#2	0.8549	0.8966	0.9764	0.9997	0.9607	0.9723	0.2129	0.9937
	#3	0.8473	0.7607	0.9044	0.9964	0.8923	0.7685	0.9251	0.9952
	#4	0.6923	0.7071	0.6553	0.6688	0.9646	0.7411	0.9975	0.8892
	#5	0.9709	0.7379	0.9702	0.9833	0.9623	0.7254	0.9970	0.9991
$R^{2}$	#6	0.8887	0.8395	0.8923	0.9074	0.7181	0.8160	0.8990	0.9007
	#7	0.9246	0.8313	0.8561	0.9973	0.9524	0.7987	0.9677	0.9715
	#8	0.9321	0.7395	0.9995	0.9997	0.6444	0.8160	0.9909	1.0000
	#9	0.9069	0.8010	0.9967	0.9981	0.9658	0.7368	0.9989	0.9990
	#10	0.8003	0.8245	0.9252	0.9174	0.8155	0.7646	0.7833	0.9946

## Data Availability

Data are contained within the article.

## References

[1] Smith I., Mackay J., Fahrid N., Krucheck D. (2011). Respiratory rate measurement: A comparison of methods. Br. J. Healthc. Assist..

[2] Favilla R., Zuccalà V.C., Coppini G. (2019). Heart rate and heart rate variability from single-channel video and ICA integration of multiple signals. IEEE J. Biomed. Health Inf..

[3] Kong W., Dong Z.Y., Jia Y., Hill D.J., Xu Y., Zhang Y. (2019). Short-Term Residential Load Forecasting Based on LSTM Recurrent Neural Network. IEEE Trans. Smart Grid.

[4] Wang Q., Zhang Y., Chen G., Chen Z., Hee H.I. (2021). Assessment of heart rate and respiratory rate for perioperative infants based on ELC model. IEEE Sens. J..

[5] Wang Q., Lyu W., Cheng Z., Yu C. (2023). Noninvasive Measurement of Vital Signs with the Optical Fiber Sensor Based on Deep Learning. J. Light. Technol..

[6] Shin Y.-S., Kim J. (2023). Sensor Data Reconstruction for Dynamic Responses of Structures Using External Feedback of Recurrent Neural Network. Sensors.

[7] Wang M., Ye X.-W., Jia J.-D., Ying X.-H., Ding Y., Zhang D., Sun F. (2024). Confining Pressure Forecasting of Shield Tunnel Lining Based on GRU Model and RNN Model. Sensors.

[8] Xie H., Huang Z., Leung F.H.F., Ju Y., Zheng Y.-P., Ling S.H. A Structure-Affinity Dual Attention-based Network to Segment Spine for Scoliosis Assessment. Proceedings of the 2023 IEEE International Conference on Bioinformatics and Biomedicine (BIBM).

[9] Zhang Q., Wang Q., Lyu W., Yu C. A Deep Learning-based Model for Human Non-invasive Vital Sign Signal Monitoring with Optical Fiber Sensor. Proceedings of the 2023 Asia Communications and Photonics Conference/2023 International Photonics and Optoelectronics Meetings (ACP/POEM).

[10] İşbitirici A., Giarré L., Xu W., Falcone P. (2024). LSTM-Based Virtual Load Sensor for Heavy-Duty Vehicles. Sensors.

[11] Boostani R., Karimzadeh F., Nami M. (2017). A comparative review on sleep stage classification methods in patients and healthy individuals. Comput. Methods Programs Biomed..

[12] Toso F., Milanizadeh M., Zanetto F., Grimaldi V., Melloni A., Sampietro M., Morichetti F., Ferrari G. Self-Stabilized Silicon Mach-Zehnder Interferometers by Integrated CMOS Controller. Proceedings of the 2021 IEEE 17th International Conference on Group IV Photonics (GFP).

[13] Dar M.N., Akram M.U., Khawaja S.G., Pujari A.N. (2020). CNN and LSTM-Based Emotion Charting Using Physiological Signals. Sensors.

[14] Specht D.F. (1991). A general regression neural network. IEEE Trans. Neural Netw..

[15] Wang Q., Lyu W., Zhou J., Yu C. (2023). Sleep condition detection and assessment with optical fiber interferometer based on machine learning. iScience.

[16] Vijayasankar A., Kumar P.R. Correction of blink artifacts from single channel EEG by EMD-IMF thresholding. Proceedings of the 2018 Conference on Signal Processing and Communication Engineering Systems (SPACES).

[17] McNicholas W.T. (2018). Comorbid obstructive sleep apnoea and chronic obstructive pulmonary disease and the risk of cardiovascular disease. J. Thorac. Dis..

[18] Sun L., Huang S., Li Y., Gu C., Pan H., Hong H., Zhu X. (2020). Remote Measurement of Human Vital Signs Based on Joint-Range Adaptive EEMD. IEEE Access.

[19] Wolpert E.A. (1969). A manual of standardized terminology, techniques and scoring system for sleep stages of human subjects. Electroencephalogr. Clin. Neurophysiol..

[20] Landry G.J., Liu-Ambrose T. (2014). Buying time: A rationale for examining the use of circadian rhythm and sleep interventions to delay progression of mild cognitive impairment to Alzheimer’s disease. Front. Aging Neurosci..

[21] Sezer E., Işik H., Saracoğlu E. (2010). Employment and comparison of different artificial neural networks for epilepsy diagnosis from eeg signals. J. Med. Syst..

[22] Ulina M., Purba R., Halim A. Foreign Exchange Prediction using DEMA and Improved FA-LSTM. Proceedings of the 2020 Fifth International Conference on Informatics and Computing (ICIC).

[23] Zhang P., Wang M. (2021). Variation Characteristics Analysis and Short-Term Forecasting of Load Based on DEMA. Proceedings of the 2021 International Symposium on Electrical, Electronics and Information Engineering (ISEEIE 2021).

[24] H S., Venkataraman N. (2023). Proactive Fault Prediction of Fog Devices Using LSTM-CRP Conceptual Framework for IoT Applications. Sensors.

[25] Wang Q., Lyu W., Chen S., Yu C. (2023). Non-Invasive Human Ballistocardiography Assessment with the Optical Fiber Sensor Based on Deep Learning. IEEE Sens. J..

[26] Wang Z., Juhasz Z. (2023). GPU Implementation of the Improved CEEMDAN Algorithm for Fast and Efficient EEG Time–Frequency Analysis. Sensors.

[27] Wang X., Nie D., Lu B. (2011). Eeg-based emotion recognition using frequency domain features and support vector machines. Lect. Notes Comput. Sci..

[28] Piuzzi E., Pisa S., Pittella E., Podestà L., Sangiovanni S. (2020). Wearable Belt with Built-In Textile Electrodes for Cardio—Respiratory Monitoring. Sensors.

